# Supplementary material for: Novel Driver Strength Index highlights important cancer genes in TCGA PanCanAtlas patients
Source: PeerJ. 2022 Aug 11;10:e13860. doi: 10.7717/peerj.13860 (PMC9375969; doi:10.7717/peerj.13860)
Supplement: Supplemental Information 2 [file peerj-10-13860-s002.zip › COHORTS/genes plots/cohorts/2021_8_16_14_9_distribution_cohorts_COAD.pdf]

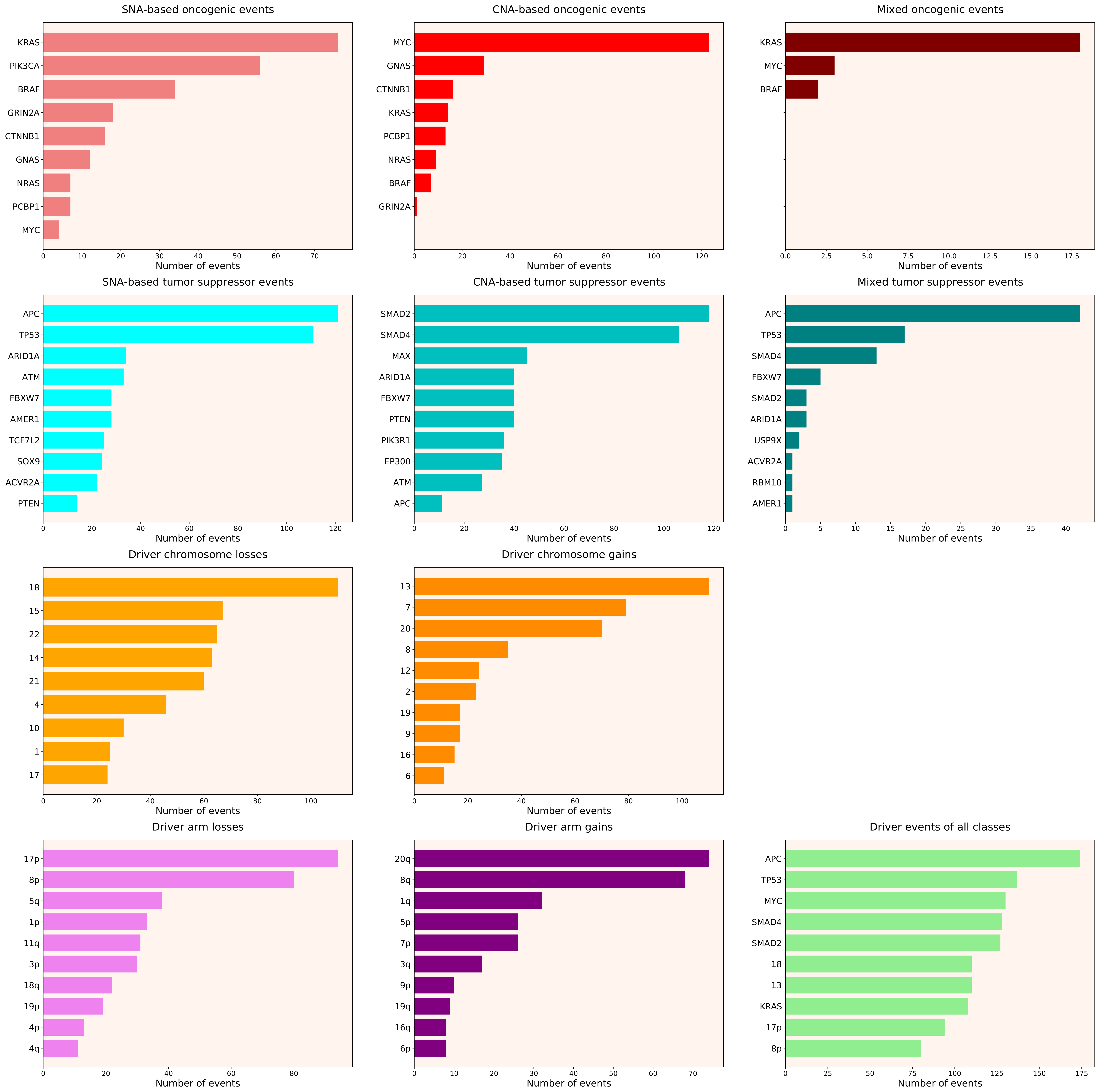

SNA-based oncogenic events
KRAS
PIK3CA
BRAF
GRIN2A
CTNNB1
GNAS
NRAS
PCBP1
MYC
0 10 20 30 40 50 60 70
Number of events
CNA-based oncogenic events
MYC
GNAS
CTNNB1
KRAS
PCBP1
NRAS
BRAF
GRIN2A
0 20 40 60 80 100 120
Number of events
Mixed oncogenic events
KRAS
MYC
BRAF
0.0 2.5 5.0 7.5 10.0 12.5 15.0 17.5
Number of events
SNA-based tumor suppressor events
APC
TP53
ARID1A
ATM
FBXW7
AMER1
TCF7L2
SOX9
ACVR2A
PTEN
0 20 40 60 80 100 120
Number of events
CNA-based tumor suppressor events
SMAD2
SMAD4
MAX
ARID1A
FBXW7
PTEN
PIK3R1
EP300
ATM
APC
0 20 40 60 80 100 120
Number of events
Mixed tumor suppressor events
APC
TP53
SMAD4
FBXW7
SMAD2
ARID1A
USP9X
ACVR2A
RBM10
AMER1
0 5 10 15 20 25 30 35 40
Number of events
Driver chromosome losses
18
15
22
14
21
4
10
1
17
0 20 40 60 80 100
Number of events
Driver chromosome gains
13
7
20
8
12
2
19
9
16
6
0 20 40 60 80 100
Number of events
Driver arm losses
17p
8p
5q
1p
11q
3p
18q
19p
4p
4q
0 20 40 60 80
Number of events
Driver arm gains
20q
8q
1q
5p
7p
3q
9p
19q
16q
6p
0 10 20 30 40 50 60 70
Number of events
Driver events of all classes
APC
TP53
MYC
SMAD4
SMAD2
18
13
KRAS
17p
8p
0 25 50 75 100 125 150 175
Number of events
